# Exploring Self‐Report Dietary Assessment Tools Validated for Indigenous Populations Globally: A Scoping Review

**DOI:** 10.1002/hpja.70038

**Published:** 2025-04-08

**Authors:** Melissa Kilburn, Yvonne Hornby‐Turner, Dympna Leonard, Valda Wallace, Sarah G. Russell, Rachel Quigley, Edward Strivens, Rebecca Evans

**Affiliations:** ^1^ College of Medicine and Dentistry James Cook University (JCU), Nguma‐Bada Campus Cairns Queensland Australia; ^2^ Australian Institute of Tropical Health and Medicine James Cook University Cairns Queensland Australia; ^3^ Cairns and Hinterland Hospital and Health Service Cairns Queensland Australia

**Keywords:** Aboriginal and Torres Strait Islander peoples, dietary assessment, dietary intake, dietary measurement, indigenous peoples, validity and reliability

## Abstract

**Issue Addressed:**

Health promotion for Indigenous populations commonly centres around diet‐mediated chronic diseases and is often evaluated with self‐report (personal recall)‐based tools. Accurate dietary assessment methods are crucial for the evaluation of these health promotion outcomes. Dietary assessment tools may require cultural, contextual and language adaptation, as well as validation within Indigenous populations to ensure efficacy and reliability. Due to the limited literature available for Aboriginal and Torres Strait Islander peoples, this review aimed to explore the range of self‐report dietary assessment tools that have undergone validation or reliability testing for Indigenous adult populations globally and their adherence to gold‐standard Indigenous research principles.

**Methods:**

This scoping review was conducted as per the Joanna Briggs Institute (JBI) method. Seven electronic databases were searched with no date or language restrictions. Screening, data extraction and quality appraisal with a validated Aboriginal and Torres Strait Islander research Quality Appraisal Tool (QAT) were undertaken by two reviewers, with a third reviewer engaged for resolving discrepancies.

**Results:**

Twenty‐five articles describing 31 instances of validity and reliability testing on 28 unique self‐report dietary assessment tools were included in the review. Studies were predominantly conducted in the USA (*n* = 13), followed by Australia (*n* = 4), Canada (*n* = 3) and Greenland (*n* = 3). The most common method of validation was relative validity (*n* = 23). Tools were primarily interviewer‐administered food frequency questionnaires (FFQs) validated against multiple 24‐h dietary recalls. Tools commonly assessed energy, carbohydrate, fat and protein intake; however, they achieved varying strengths of correlation (*r* = 0–0.82). Tools were predominately paper‐based; however, six studies validated a device‐based tool; no web‐browser app‐based tools were validated in the included literature.

**Conclusion:**

Interviewer‐administered food frequency questionnaires are the most prevalent self‐report dietary assessment method validated within Indigenous populations globally. Browser‐based e‐tools, which are portable and cost‐effective, may hold promise for dietary assessment among Indigenous populations. The acceptability and validity of such tools for Indigenous population groups should be explored through future research. Tools validated to capture added sugar, sodium and food group intake may provide for more meaningful evaluation of health promotion programmes for Indigenous peoples.

**So What:**

Tools that have been validated for use with Indigenous peoples are essential for supporting a reliable and accurate evaluation of health promotion activities. Validating dietary assessment tools to adequately capture the predominant outcome measures targeted in nutritional health promotion strategies within Indigenous populations may contribute a more meaningful evaluation of health promotion programmes for Indigenous peoples.

## 1 Introduction

Traditional lifestyles of Aboriginal and Torres Strait Islander peoples were inherently healthy. Diets were subsistence in nature and characterised by lean meats and plant foods, high in protein and fibre and low in fats and sugars, which necessitated regular and incidental physical activity to sustain [1]. Western colonisation and the systematic dispossession of ancestral lands, food systems and waterways from Aboriginal and Torres Strait Islander peoples resulted in abrupt and major disruptions to pre‐existing lifestyle patterns [1, 2].

Fragmentation in traditional lifestyle practices, including restricted access to lands used for hunting and gathering and the forced adoption of western foods and cooking practices has, over time, resulted in deviation away from previously innately healthy lifestyles. This disrupted the balance and co‐nurturing synergy between peoples, the lands and waterways that had been curated and optimised through generational learnings, whilst simultaneously suppressing the ability to pass down these knowledge systems to future generations [1, 2]. As a result, the lifestyles of Aboriginal and Torres Strait Islander peoples within Australia were irrevocably altered. These experiences are also mirrored in other colonised Indigenous populations including Canada, New Zealand and the USA [3].

The prevalence of non‐communicable chronic diseases such as type 2 diabetes, cardiovascular disease and dementia among Australians has increased by 38% in the last three decades [4] and whilst there has been significant financial investment in more recent years in preventative health care targeting chronic disease, these conditions remain disproportionally high for Aboriginal and Torres Strait Islander peoples [5]. Achieving health equity for Indigenous peoples requires a whole systems approach [6], from individual‐level knowledge of healthy lifestyle behaviours to infrastructure and policies that support their implementation. Health promotion programmes are uniquely positioned to operate along this spectrum as both a driver and mechanism of these health systems. Consequently, health initiatives require consistent and accurate tools to enable evaluation of targeted health outcomes. A healthy diet is one of the top protective factors against many chronic diseases and is featured heavily in health promotion programmes for Indigenous populations. As such, having culturally appropriate and relevant dietary assessment methods for these population groups is of particular importance for determining the success of programmes.

A range of dietary assessment methods exist including individual‐level tools including food frequency questionnaires (FFQs), 24‐h dietary recalls, food diaries and weighed food records, as well as community‐level methods such as the store‐turnover method. Self‐report questionnaire style tools—where the participant is reporting on their own personal dietary intake—such as FFQs, are often the tool of choice for measuring the health promotion programme outcomes for Aboriginal and Torres Strait Islander populations [7]. This is likely due to their portability, relatively low cost and ability to capture habitual dietary intake but also because many other methods have been found to be inappropriate and prohibitively burdensome when trialled with Indigenous populations [7].

Many existing dietary assessment methods are euro‐centric in design and are therefore limited in their ability to capture traditional foodways of Indigenous populations. Cultural, contextual and language considerations may also prevent these tools from performing equitably for Indigenous peoples [7, 8]. A scoping review by Davies et al. (2023) exploring dietary assessment methods used for research with Aboriginal and Torres Strait Islander peoples in Australia found that weighed food records were inappropriate due to incompatibility with cultural practices and duties and high participant burden, and that FFQs often have rigid options that do not align with local foodways and seasonal food availability for Indigenous communities [7]. Additionally, Indigenous peoples' may be bi‐ or tri‐lingual in predominantly oral‐based languages rather than written English, which imposes further caveats on the accessibility of existing dietary assessment methods for Indigenous peoples [9]. All these factors notwithstanding, the risk of social desirability bias although inherent for any population group with self‐report tools, may be exacerbated by the historical weaponisation of western research methods against Indigenous peoples as an enduring legacy of colonisation [7]. Thus, whilst the need for tools that measure dietary intake to be portable and of low participant burden persists, it is necessary to ensure that such tools have been designed and appropriately tested and validated to be contextually relevant for Indigenous peoples and communities. This can be achieved by appropriate community engagement, embedding Indigenous governance and leadership and co‐designing tools and research methods with Indigenous peoples to prioritise Indigenous understandings of health.

Validation is the process to determine that the method in question is suitably able to capture or assess the desired outcome measure, whereby reliability testing ensures that tools are consistent in their measurement [10]. Both concepts are important for establishing that a tool is accurate and reliable, particularly if the outcome measures are being used for decision making at an individual, community, or policy level. Within these two broad terms are several types of testing which would be used depending on the intention of the tool or its use thereafter, as defined by the DAPA (Diet and Physical Activity) Measurement Toolkit [10]: *Face validity and acceptability* determine whether a tool is satisfactory and relevant to the target demographic. *Content validity* aims to ensure that data collected by the tool encompasses all relevant data points and concepts. *Test–retest reliability* ensures the tool measures consistently between administrations. *Inter‐rater reliability* determines the consistency of the outcome measures between different administrators. *Concurrent or relative validity* (hereafter referred to as relative validity) determines how closely correlated the outcome measures are compared to an established reference method measuring the same outcome. *Reference method* refers to the established measure against which the novel assessment instrument is compared; ideally, gold‐standard reference methods would be utilised such as doubly labelled water (DLW) for energy expenditure and weighed food records (WFRs) and/or certain biomarkers for dietary recall methods [10], although there are contextual considerations about the applicability of gold‐standard reference methods for diverse populations. The strength of correlation between the novel tool and the reference method then determines the relative validity of the tool. No universal gold‐standard reference method exists to validate dietary assessment methods. Diet is complex and although some biomarkers exist that are good indicators of intake of some foods, there is no biomarker to assess whole diet [10]. Similarly, the most robust reference methods, such as 3‐day WFRs, can be overly burdensome and may distort participants true intake due to observer bias [7, 11]. Dietary assessment tools, such as those used in health promotion evaluation or clinical settings, are generally expected to have a comprehensive assessment of reliability and validity, including relative validity against an established reference method, from which to benchmark the accuracy of results [10].

A preliminary search was unable to locate existing review literature that consolidated the range of tools that have undergone validation among Aboriginal and Torres Strait Islander peoples or Indigenous populations globally, suggesting it is yet to be systematically assessed. Davies et al. (2023) explored the range of dietary assessment methods used for research within Aboriginal and Torres Strait Islander populations in Australia [7]. Although their review was not focused on validation studies, they reported that three of the 22 included studies in their review assessed the validity of methods utilised (store‐turnover method, 24‐h recall method and weighed food record) [7]. The lack of Australian data means it is necessary to explore the international literature on the topic, particularly for self‐report methods such as FFQs and diary methods due to their prominence in Indigenous populations health promotion evaluation.

### 1.1 Aim

This scoping review aimed to identify and examine the scope of self‐report dietary intake assessment tools and their validation or reliability testing methods for Indigenous populations globally to inform the development of a self‐report dietary assessment tool for an Indigenous population in Australia.

### 1.2 Objectives

- Identify key themes and methodological variations in tool development and validation and reliability testing for Indigenous populations globally (including description of tool characteristics and modalities of administration).
- Assess the methodological quality of these studies through an Indigenous research lens.
- Summarise the reported outcomes in dietary assessment tool validation for Indigenous population groups.

## 2 Methods

A scoping review was the most appropriate method to explore this topic given the dearth of existing review literature on the topic, and the intention to scope both the range of existing dietary assessment tools and their methods to validate for Indigenous population groups [12]. This scoping review was conducted according to the JBI manual for Scoping Reviews [13]. The protocol has been registered on Open Science Framework (doi: https://doi.org/10.17605/OSF.IO/VKUZH).

Due to the expected limited number of results on the topic, the eligibility criteria were intentionally broad.

### 2.1 Inclusion/Exclusion Criteria

Studies that included adults from Indigenous populations in any location globally were included. Studies were excluded if the validation was not specific to Indigenous peoples or, in the case of multiethnic samples, if results were not reported separately for Indigenous peoples within the sample. Studies were also excluded if the sample population was exclusively children. There were no exclusions based on participant health status or co‐morbidities. Studies that reported on any form of validity or reliability testing of self‐report dietary assessment methods [10] were included. Individual‐level dietary assessment methods such as questionnaires, surveys and device applications, conducted in any setting: phone, community, hospital or other health care provider, were included. Dietary assessment methods including whole diet, specific foods and alcohol were included. Community‐level data collection such as store turnover method was excluded.

### 2.2 Data Sources

This review considered all primary data sources that reported on the validity or reliability of dietary assessment tools within Indigenous populations globally. Review literature, protocols and conference abstracts were excluded.

The search strategy was developed by MK and YHT with consultation from University Information Specialists (JC & SA). The search query included terms that described any validation process of self‐report dietary assessment methods, as defined elsewhere [10], in any Indigenous population globally (Appendix A). Seven electronic databases were searched in February 2023 including Medline, EmCare, CINAHL, PsychInfo, Scopus, Web of Science, Australian Indigenous Health InfoNET as well as the first 50 results from an advanced search of Google Scholar. No date or language restrictions were imposed. The reference lists of all eligible studies were also screened for additional eligible articles. Database search alerts were enabled for the duration of the review period in all databases except for Google Scholar and Australian Indigenous Health InfoNET, for which searches were duplicated in May 2024 to capture any articles published since the initial searches were performed. Search results and review inclusion/exclusion results were reported according to the PRISMA extension for scoping reviews reporting guidelines (PRISMA‐Scr) and mapped in a PRISMA flowchart [14].

Search results were imported into EndNote 20 (Philadelphia, PA: Clarivate; 2013) and duplicates were removed. Title and abstract screening and full text screening were independently performed in Excel against the eligibility criteria by two reviewers (MK and DL), with reasons for exclusion recorded. Discrepancies were discussed and resolved between the two reviewers.

### 2.3 Data Extraction

Data were extracted and charted within Excel, utilising a modified version of the JBI data extraction form [13]. Additional fields were included relevant to the research questions, with input from all authors. The charted data (Table 1) included: study location, population, sample size, tool characteristics (method of administration, number of items, diet/food domains assessed, recall period, tool novelty (pre‐existing vs. newly developed), use of translation or interpreters). Tool validation methods were also extracted including type of validation (content, relative, construct), reference methods used (if applicable) and reported validation outcome measures. Data extraction and charting was completed by the lead author (MK) and duplicated for 30% of articles by the second reviewer (DL) with comparison by both reviewers and consensus that data extraction parity was achieved.

**TABLE 1:** Summary of characteristics of studies validating self‐report dietary assessment methods in Indigenous populations globally.

Article #	Study			Assessment tool		Validation		
	References	Population	Aims/Purpose/Rationale	Tool (name, type, characteristics)	Intake domains assessed	Type of validation	Validation reference method	Summary of validation findings
18	Abu‐Saad et al. [15]	*n* = 60 Aboriginal adults, 70% women aged 42–55 years Bunbury NSW and Perth WA, Australia	To perform pilot assessment of a digital FFQ for use in the Kaat Koort programme for Indigenous adults in Australia *Rationale for tool validation*: Chronic disease prevention	Meal‐Based FFQ Newly developed tool 81 main items Extra options available > 300 items 30‐day recall period Interviewer‐administered (Dietitian), entered into I‐ACE software (requires training) English language	Core Food Groups Energy (EEI) (kJ) **Macronutrients** Fat: [total] (g) Carbohydrate: [total, sugar, free sugar] (g) Protein [total] (g) Fibre (g) **Micronutrients** Ca (mg) K (mg) Na (mg) Mg (mg)	Face validity/acceptability	Comparison of EEI with EEE % of main food list that contributed to overall intake	EEI from tool (10042kj/day) similar to EEE(10197kj/day) 66%–90% participants reported nutrient and energy intakes were foods from main list.
19	Andersen et al. [16]	*Multiethnic sample* *n* = 535 adults Male and Female 434 Inuit 101 non‐Inuit Aged 50–69 years Across four Greenland population groups Inuit living in metro, regional and rural areas and non‐Inuit	‘To ascertain iodine intakes, factors affecting iodine intake in circumpolar populations, and the usefulness of urinary iodine excretion as a biomarker for validation of Inuit food‐frequency questionnaires’ *Rationale for tool validation*: Iodine deficiency	FFQ Newly developed tool 14‐item 7 traditional food items 7 imported food items 6 frequency categories Never—Daily 12‐month recall period Interviewer‐administered Choice of Danish or Greenlandic with Interpreter Traditional foods were scored positively and imported food negatively to give overall score, which then classified intake level	Traditional Inuit food intake score Seasonal variation	Relative validity	Interviewer‐administered Survey (2‐item cross‐check) at the time of tool administration Biomarker (Iodine – spot urine samples) at tool administration and sub‐sample 24‐h collection	Inuit food frequency correlated well between the cross‐check questions and FFQ (Kendall's tau 0.8, *p* < 0.001). Median urinary iodine excretion declined with the degree of decrease in the traditional lifestyle and increase in imported foods (*p* < 0.001).
20	Ashman et al. [17]	*Multi‐ethnic sample n* = 25 pregnant adult women (8 Aboriginal women, 17 non‐Indigenous women) Aged 20–50 years NSW Australia	‘(1) To assess the relative validity of image‐based dietary records from assessment of intake of Indigenous and non‐Indigenous Australian pregnant women against 3× 24‐h recalls. (2) Assess the inter‐rater reliability between two independent dietitians in assessing 3‐day image‐based dietary records in a sub‐sample of participants (*n* = 10). (3) Assess the quality of image‐based dietary records and voice/text description for analysis. (4) Assess the perceived useability and acceptability of the image‐based dietary records’ *Rationale for tool validation*: Optimal pregnancy nutrition	SNaQ Newly developed tool 3‐day image‐based dietary record Self‐administered via uploading images of all meals and snacks from smartphone. Dietitian assessed nutritional analysis of uploaded foods by entering into nutritional software with option to include voice memo or text notes English language	Energy (kJ) **Macronutrients** Protein [total] (g) Fat [total, saturated] (g) Carbohydrate [total] (g) Fibre (g) **Micronutrients** Ca (mg) I (mg) Fe (mg) K (mg) Mg (mg) Na (mg) Zn (mg) *Vitamins* D (μg) E (mg) Folate (μg)	Face validity/Acceptability Inter‐rater reliability Relative validity	Survey adherence Nutrient analysis by 2× independent Dietitian 3× 24‐h dietary recalls (telephone and in‐person) Randomised days in 3 weeks post SNaQ data collection	88% total sample and 100% Indigenous portion of sample reported tool acceptable. Intraclass coefficients EEI: 0.929 (*p* < 0.001) Macronutrients 0.865–0.932 (*p* < 0.05): Fat, Protein, Carbohydrate, Fibre Micronutrients 0.794–0.988 (*p* < 0.05): Folate, Iron, Iodine, Calcium, Zinc *The relative validity findings were not reported separately for Aboriginal participants for the so this component was excluded from this review*
21	Baer et al. [18]	*Multi‐ethnic sample* *n* = 279 pregnant women, aged 16–40 years 104 American Indian/Alaska Native—Spirit Lake Sioux and Turtle Mountain Chippewa 175 Caucasian North Dakota, USA	‘To examine the validity of the Harvard Service FFQ (HSFFQ) among low‐income American Indian and Caucasian pregnant women’ *Rationale for tool validation*: Optimal pregnancy nutrition	Harvard Service FFQ (HSFFQ) Pre‐existing tool 100‐item 4‐week recall period Self‐administered paper form English language Adapted to include culturally relevant foods	Energy (kcal) **Macronutrients** Carbohydrate [total, sugar] (g) Fat [total, saturated, monounsaturated, polyunsaturated, omega 3 fatty acids] (g) Protein [total] (g) Cholesterol (mg) Fibre (g) **Micronutrients** Ca Fe Mg P Zn (mg) *Vitamins* A (IU) B1 (mg) B2 (mg) B6 (mg) B12 (mg) Folate (μg)	Concurrent validity	3× 24‐h dietary recalls over 1 month	Lower correlation coefficients for American Indian/Alaska Native women than the Caucasian women. CI for the correlations between the tool and mean of the 24 h recalls for American Indian/Alaska Native women were wide and, in several cases, crossed over the no effect line EEI *r* = 0.15 (CI = −0.13–0.41)—similar results with Fibre, Sucrose, polyunsaturated fat and omega 3 fatty acids, Otherwise, Pearson correlations ranged from *r* = 0.35–0.73 Macronutrients: *r* = 0.15–0.62 Minerals: *r* = 0.35–0.55 Vitamins: *r* = 0.43–0.73
22	Bjerregaard et al. [19]	*n* = 5757 Inuit participants Aged 15 years and older Male and Female not otherwise specified Greenland National Sample	‘To compare information on the consumption of tobacco and alcohol obtained from three population surveys in Greenland with import statistics’ *Rationale for tool validation*: National survey instrument requires validation	4× different pre‐existing alcohol intake surveys that formed part of historical National Health surveys: Self‐administered Translated and back translated (Danish—Greenlandic) Last occasion: – 2 items: when last had an alcoholic beverage and amount Weekly days – 3 items: frequency, weekly intake and amount on last occasion Beverage specific – 3 items: habitual intake of beer, wine and spirits Weekly + Binge – Combination of weekly days instrument above and binge frequency (used assumed binge intake amount to calculate overall intake)	Alcohol intake (L/year) (average reported intake from National Health Survey participants extrapolated out to national adult population)	Relative validity	Alcohol Import Statistics (L/year) (population level)	Correlation using General Linear Model Weekly + Binge method accounted for 78% of import statistics from same period however the other methods each ranged between 40% and 51% of imports indicating gross under reporting
23	Bjerregaard et al. [20]	91 Inuit adults Greenland Male (41.8%) and Female (58.2%) Aged 26–78 years from three settlements—Metro: Nuuk, Regional: Aasiaat and rural: Attu	‘To evaluate the reproducibility of the revised 45‐item FFQ among the Inuit in Greenland, to validate it by comparison to a food diary and to compare its questions about locally harvested food with whole blood mercury as a biomarker’ *Rationale for tool validation*: National survey instrument requires validation	FFQ Frequency with serve size quantification Newly developed tool (revised previously used National tool being validated for the first time) 45‐item 12‐month recall period Interviewer‐administered Danish language with Greenlandic language interpreter	Core Food Groups Energy (kJ) **Macronutrients** Carbohydrate [total, sugar] (g) Fat [total, polyunsaturated, EPA, DHA] (g) Protein [total] (g) Fibre (g) Mercury (μg)	Relative validity Test retest reliability	Diary (*t*‐test) daily for 3 weeks across 3 different seasons Biomarker Mercury (Spearman Correlation) Tool readministered 41 months post initial administration	Significant differences between FFQ and Food Diary *p* < 0.0001 Specifically for Food Groups: Fruit and Veg, Dairy, Grains, Beverages Significant correlations for all foods assessed (*p* < 0.0001) Marine Mammals *r* = 0.2–0.5 (*p* < 0.0001) Fish: *r* = 0.23–0.27 Mercury intake: *r* = 0.38–0.49 Blandt–Altman plots show high agreement between the two FFQ administrations
24	Edwards et al. [21]	*n* = 2946 Navajo adults, aged 18–69 years 35% male and 64% female Southwest, USA	Not reported *Rationale for tool validation*: Explore new technologies for dietary assessment	EARTH DHQ Newly developed Diet History Questionnaire Frequency with serve size quantification 12‐month recall period Self‐administered via computer English language with audio translation	Not reported	Face validity/acceptability	Usage data Usability survey: Self‐administered 14‐item paper survey. Likert scale responses on ease of use and preferences	Low use of HELP button (7.8%) 71.4% used the audio all of the time 36‐min average completion duration High acceptability (96%) despite low personal computer usage of participants, increased with education level Age inversely related to useability
25	Fialkowski et al. [22]	*n* = 525 American Indian/Alaska Native adults from three Tribal Nations in Washington State, USA Male (42%) and Female (58%) Mean age: 41.5/43 (M/F)	‘To evaluate three dietary assessment tools: dietary records, a food frequency questionnaire (FFQ), and a shellfish assessment survey among Native American adults form Communities Advancing the Studies of Tribal Nations Across the Lifespan (CoASTAL) cohort’. *Rationale for tool validation*: Chronic disease prevention	**Block FFQ:** Pre‐existing scannable paper tool Frequency with pictures for serve size quantification Number of items not reported 12‐month recall period Self or Interviewer‐administered depending on literacy English language **Shellfish questionnaire:** Adapted pre‐existing tool to include locally/culturally relevant foods Recall of most recent intake Self‐administered Number of items not reported, 10–15 min to complete English language	Core Food Groups Energy (kcal) **Macronutrients** Carbohydrate [total] (g) Fat [total, saturated, trans, monounsaturated, polyunsaturated] (g) Protein [total] (g) Cholesterol (mg) Fibre (g) **Micronutrients** Ca (mg) Fe (mg) *Vitamins* C (mg) Folate (μg) Intake of: Seafood Shellfish Clams Chowder	Relative validity	2× 1‐ and 1× 2‐day dietary records taken at 4‐month intervals over 12 months Non‐consecutive days, weekday and weekend	Pearson correlations between FFQ and dietary records were not significant for total EEI or Fat Significant correlations reported for % energy from protein (*r* = 0.45), and carbohydrate (*r* = 0.39–0.50). *Micronutrients*: significant correlations for Ca (*r* = 0.45–0.51), other findings were not statistically significant. Significant Spearman correlations between shellfish questionnaire and the FFQ for reported seafood and shellfish intake (*r* = 0.13–0.20, *p* < 0.001) however not significant for correlation to dietary records.
26	Hankin et al. [23]	*Multi‐ethnic sample* *n* = 262 47 Native Hawaiians 47 Filipino 55 Caucasian 58 Japanese 55 Chinese Random sample from Hawaii State Department of Health, Health Surveillance Programme Male (49%) and Female (51%) Aged 45–75 years Hawaii, USA	To evaluate the diet history method accompanied by photographs for portion size quantification as a comprehensive measure of usual food and nutrient intakes of adults living in Hawaii *Rationale for tool validation*: Chronic disease prevention	Diet History Questionnaire (DHQ) Newly developed Frequency with serve size quantification 47‐item 12‐month recall period Interviewer‐administered English language	**Macronutrients** Fat [total, saturated, polyunsaturated] (g) Protein [total] (g) Cholesterol (mg) *Vitamins* A (IU) Beta‐carotene (μg) C (mg)	Relative validity	4× 7‐day food diaries each 3‐month interval over 12 months	Weaker ICC correlations for all aspects for Native Hawaiians than the other ethnic groups ICC: 0.31–0.47
27	Hartman et al. [24]	*n* = 164 Northern Plains American Indian/Alaska Native pregnant women with a mean age of 27 years USA	‘To administer a short dietary screener to pregnant women to estimate intakes of selected food groups and to assess the relative validity of the screener compared with similar measures obtained from repeat 24‐h dietary recalls’ *Rationale for tool validation*: Optimal pregnancy nutrition	PASS Dietary Screener Newly developed 44‐item self‐administered survey Assesses usual intake Frequency with serve size quantification and preparation methods Culturally relevant foods included English language	Core Food Groups **Macronutrients** Carbohydrate [added sugar] (g) Fat [saturated fat, discretionary solid fat] (g) **Micronutrients** Na (mg)	Relative validity	3× 24‐h dietary recalls via phone within 4 weeks of initial tool administration Randomly selected, non‐consecutive days	Pearson Correlation coefficients between the PASS screener and average of the 24 h recalls for each food groups ranged from *r* = 0.38–0.72 with majority of food groups being 0.4 or higher, all statistically significant (*p* < 0.05) except for the meat, fish and poultry group with *r* = 0.31 (*p* = 0.169)
28	Kolahdooz et al. [25]	*n* = 177 Yup'ik males (*n* = 73) and females (*n* = 104) aged 13–88 years from six western Alaska communities USA	‘To develop a quantitative food frequency questionnaire to assess the diet of the Yup'ik people of western Alaska’ *Rationale for tool validation*: Chronic disease prevention	Quantitative Food Frequency Questionnaire (QFFQ) Newly developed tool Frequency with serve size quantification 150‐item 12‐month recall period Interviewer‐administered paper tool English language Local language interpreter	Not reported	Face validity/acceptability	Pilot testing (*n* = 15)	Confirmed cultural appropriateness and usability of tool. Required modification by grouping similar items to reduce time burden on participants
29	Lee et al. [26]	*n* = 41 Aboriginal adults Northern Coastal and Central Desert communities not further specified Australia	‘To identify a practical quantitative dietary survey methodology acceptable to remote Aboriginal communities, to assess the face validity of each method and to compare the qualitative data obtained’ *Rationale for tool validation*: Chronic disease prevention	FFQ Pre‐existing tool Assesses usual intake Interviewer‐administered English language with local language interpreter Modified to include culturally/locally relevant foods Not further specified	Authors report no quantitative data produced	Face validity	Not explicitly reported, appears researchers rated the features of the methods based on observations, does not state if participants contributed to this assessment	Scoring for assessment items: **Very low:** Lack of individual data, acceptance to community, retrospective data, suits Aboriginal involvement, potential reproducibility **Low**: intrusiveness, resources required, measuring usual diet, cost effectiveness **Moderate**: time, personnel, travel costs
30	Lee et al. [27]	*n* = 206 Aboriginal and Torres Strait Islander adult males (51.6%) and females, aged 18–78 years, from primary health facilities in QLD and SA Australia	To assess the validity of the Finnish method [for assessing alcohol intake] as delivered by the Grog app with Aboriginal and Torres Strait Islander Australians *Rationale for tool validation*: improve alcohol intake assessment for Indigenous Australians	Grog App Newly developed survey tool Recall of last four drinking occasions 13‐item Self‐administered via provided tablet English language Audio recordings and translation of questions	Alcohol Intake (Standard Drinks)	Relative validity Test–retest reliability	Clinical interview Grog app readministered within 1 week of initial administration	Pearson correlation coefficients of reported alcohol intake between App and interview *r* = 0.52–0.72 (*p* < 0.01) Average consumption of alcohol was well correlated between the two administrations with Spearman correlations of *r* = 0.81.
31	Metcalf et al. [28]	*Multi‐ethnic Sample* *n* = 176 New Zealand adults, male (68.8%) and female, aged 40–65 years 124 European 18 Māori 34 Pacific Islander New Zealand	To examine the reproducibility and validity of a FFQ in the New Zealand multiethnic population *Rationale for tool validation*: Chronic disease prevention	FFQ Frequency with serve size quantification Pre‐existing tool, modified to include Polynesian foods 142‐item 3‐month recall period Self‐administered paper tool English language	Core Food Groups Energy (MJ) **Macronutrients** Carbohydrate [total] (g) Fat [total, saturated, monounsaturated, polyunsaturated] (g) Protein [total] (g) Fibre (g) **Micronutrients** Ca (mg) Alcohol Intake (g)	Relative validity Test–retest reliability	3‐day food diary within 1 week after tool administration Tool readministered 3 years apart	Nutrients: In Māori cohort, correlations between tool and 3‐day food diary were not statistically significant, apart from polyunsaturated fat intake. Pacific Islander cohort had statistically significant mean comparisons for fibre, total fat, saturated fat and cholesterol When comparing European to Māori and Pacific Islander cohorts combined there were statistically significant Spearman Correlation coefficients for: Alcohol (*r*: 0.56) and Carbohydrate % total intake (*r* = 0.39), Otherwise, findings were not significant, and correlation coefficients ranged from 0.36 to 0.63 Paired *t*‐test showed no significant differences between in the mean nutrient intakes between the two FFQ administrations for Māori and pacific Islander participants, there was an increase in polyunsaturated fat intake for Europeans that was significant however (*p* < 0.05).
32	Murtaugh et al. [29]	*n* = 124 American Indian/Alaska Native and Alaskan Native adult women, aged 35–49 years participating in the EARTH study Alaska, Dakotas, Southwest USA	‘To assess reliability and relative validity of a self‐administered computer‐assisted dietary history questionnaire (DHQ) for use in a prospective study of diet, lifestyle and chronic disease in American Indians in the Dakotas and Southwestern US and Alaska Native people’ *Rationale for tool validation*: Chronic disease prevention	EARTH DHQ Pre‐existing tool 70+ items (54 main food group questions with questions on preparation method) Pictorial guide for serve size quantification using default values on serve size 12‐month recall period Self‐administered via computer programme in English language with audio translation into local language Included culturally relevant foods	Core Food Groups [Red meat, fruit, vegetables] Energy (kCal) **Macronutrients** Carbohydrate [total, sucrose density] (g, g/1000 kcal intake) Fat [total] (g) Protein [total] (g) Fibre (g) **Micronutrients** Ca (mg) *Vitamin Density* (*mg/1000 kcal intake*) A D E	Test–retest reliability Relative validity	Re‐administration of tool within 1 month post initial administration 4× 24‐h dietary recalls via telephone over 12 months Non‐consecutive days, weekday and weekend	Pearson correlation coefficients *r* = 0.43–0.92 Spearman correlation coefficients: *r* = 0.44–0.82 Greatest for Energy and Carbohydrate intake *p*‐values not reported Pearson correlation coefficients Energy: *r* = 0.09 Macronutrients: *r* = 0.03–0.30 Vitamin density: *r* = 0.22–41 Calcium density: *r* = 0.59 Food Groups: *r* = 0.22–0.67
33	Neuhouser et al. [30]	*Multiethnic sample* *n* = 413 women 102 American Indian/Alaska Native (mean age = 38.7 years) 157 Hispanic (mean age = 38.8 years) 154 non‐Hispanic white (mean age = 31.6 years) Lower Yakima Valley Washington USA	‘To examine the use if a household food inventory as a proxy of fruit and vegetable consumption among and ethnically diverse sample of women residing in a resource‐poor region of rural Washington state and whether the inventory performs better than other instruments in relation to blood biomarkers of fruit and vegetable intake’ *Rationale for tool validation*: Chronic disease prevention	**Household Pantry Inventory** Newly developed inventory tool Yes/No comparison of inventory list to items that were present in respondent's pantry Yes = 1 point score totalled at end Interviewer‐administered Cultural Foods English/Spanish Language **5 a day screening tool** FFQ Pre‐existing tool 6‐item 1‐month recall period Interviewer‐administered English/Spanish Language	Tally Score increased by each relevant item in food storage Frequency of fruit and vegetable consumption Estimated daily servings calculated via algorithms	Relative validity Relative validity	Fasting Blood biomarkers Fasting Blood biomarkers	Pearson Correlation Coefficients between biomarkers and inventory score Total carotenoids: *r* = 0.06 (not significant) Alpha Carotene: *r* = 0.2 (*p* < 0.05) Beta‐carotene: *r* = 0.24 (*p* < 0.05) Beta cryptoxanthin: *r* = 0.18 (not significant) Lycopene: *r* = −0.02 (not significant) Lutein + zeaxanthin: *r* = 0.02 (not significant) Pearson correlation coefficients Total carotenoids: *r* = −0.03 (not significant) Alpha Carotene: *r* = 0.06 (not significant) Beta‐carotene: *r* = 0.04 (not significant) Beta cryptoxanthin: *r* = 0.03 (not significant) Lycopene: *r* = −0.12 (not significant) Lutein + zeaxanthin: *r* = −0.16 (not significant)
34	Nobmann [31]	*n* = 65 Yu'pik Adults 29 Men, 36 Women aged 40–87 years from Gambell community on St Lawrence Island Northwest Alaska USA	‘To describe the intakes of dietary factors related to heart disease, especially fats, among a homogenous group of Alaska Natives and correlate the dietary factors with physiological‐risk factors for heart disease’ *Rationale for tool validation*: Chronic disease prevention	Native Dietary Intake Questionnaire FFQ Newly Developed Frequency with serve size quantification using participants own utensils 12‐month recall period Interviewer‐administered English language with local language interpreter	Energy (kJ, kcal) **Macronutrients** Carbohydrate [total, sucrose] (g) Fat [total, saturated, monounsaturated, polyunsaturated, myristic, stearic, linoleic, EPA, omega 3, omega 6] (g) Protein [total] (g) Cholesterol (mg) Fibre [total, water soluble] (g) **Micronutrients** Fe (mg) Na (mg) Se (μg) *Vitamins* Beta‐carotene (μg) B6 (mg) B12 (μg) E (mg) C (mg) Folate (μg)	Relative validity	1× 24‐h dietary recall performed in home contemporaneous with FFQ administration	Low to Moderate Pearson Correlation coefficients for all domains *r* = 0–0.49
35	Pakseresht et al. [32]	*n* = 75 Inuit adults who were identified as the main ‘shopper’ in the family 92% female Mean age 44/45 (M/F) Nunavut, Canada	‘To assess the relative validity of the 150‐item QFFQ to estimate mean daily intake of energy, macronutrients and some micronutrients of interest, comprising those that were determined to be important for targeting in a nutrition intervention programme in this population, by comparing with repeated 24‐h recalls’ *Rationale for tool validation*: Chronic disease prevention	QFFQ Newly developed tool 150‐item 30‐day recall period Frequency with serve size quantification Interviewer‐administered English language with local language interpreter Included culturally relevant foods	Energy (MJ) **Macronutrients** Carbohydrate [total, sugar] (g) Fat [total] (g) Protein [total] (g) Fibre (g) **Micronutrients** Ca (mg) Fe(mg) Z (mg) *Vitamins* A (μg) B2 (mg) B6 (mg) B12 (μg) C (mg) D (IU) E (IU) Folate (μg)	Relative validity	Up to 3× 24‐h recalls Non‐consecutive days (weekend and weekday) Analysis performed as whole group and split into < 50 years and > 50 years	Overall: Significant de‐attenuated Spearman correlations for all measures except Iron intake. Energy: *r* = 0.65 Macronutrients: *r* = 0.28–0.74 Vitamins: *r* = 0.33–0.69 Minerals: *r* = 0.22–0.50 Smaller crude, de‐attenuated and energy‐adjusted correlation coefficients for participants > 50 years compared to participants < 50 years (*r* = 0.35, 0.37 and 0.24 vs. *r* = 0.43, 0.47 and 0.35, respectively) < 50 years significant de‐attenuated Spearman correlations all measures except Protein, Vitamins A and D Energy: *r* = 0.57 Macronutrients: *r* = 0.30–0.69 Vitamins: *r* = 0.21–0.58 Minerals: *r* = 0.36–0.51 > 50 years de‐attenuated Spearman correlations were not significant for most measures except total sugar, Vitamin A, B2, B6, B12, C, E Energy: *r* = 0.27 Macronutrients: *r* = 0.24–0.31 Vitamins: *r* = 0.02–0.73 Minerals: *r* = 0.20–0.31
36	Pakseresht et al. [33]	*n* = 64 randomly selected Inuvialuit adults who were identified as the main ‘shopper’ of the household 80% women Mean age 45/46 (M/F) Canada	‘To establish the validity of a 142‐item quantitative FFQ (QFFQ) developed to assess dietary intake in a population living in the Northwest Territories, Canada and undergoing rapid nutrition transition’ *Rationale for tool validation*: improve FFQ efficacy for Inuit	QFFQ Pre‐existing tool (refer to article # 39 above) 142‐item 30‐day recall period Frequency with serve size quantification Interviewer‐administered English language with local language interpreter Included culturally relevant foods	Energy (MJ) **Macronutrients** Carbohydrate [total, sugar] (g) Fat [total] (g) Protein [total] (g) Fibre (g) **Micronutrients** Ca (mg) Fe (mg) Z (mg) *Vitamins* A (μg) B2 (mg) C (mg) D (IU) Folate (μg)	Relative validity	Up to 3× 24‐h dietary recalls Non‐consecutive days (weekend and weekday) Analysis performed as whole group and split into < 50 years and > 50 years	Overall: Significant de‐attenuated Spearman correlations for all measures except Folate, Vitamin A, Ca, Zn Energy: *r* = 0.46 Macronutrients: *r* = 0.33–0.57 Vitamins: *r* = 0.18–0.53 Minerals: *r* = 0.19–0.37 Higher de‐attenuated Spearman correlations for the younger age groups than the > 50 years group. < 50 years significant de‐attenuated Spearman correlations for all macronutrients, Vitamins B2, C and Fe Energy: *r* = 0.61 Macronutrients: *r* = 0.48–0.67 Vitamins: *r* = 0.28–0.63 Minerals: *r* = 0.26–0.51 > 50 years de‐attenuated Spearman correlations were not significant any measures Energy: not calculated Macronutrients: *r* = 0.09–0.35 Vitamins: *r* = 0.14–0.26 Minerals: *r* = 0.25
37	Ratelle et al. [34]	*n* = 36 adult Dene and Métis adults including males, females, Elders and local harvesters not further specified Canada	‘To refine and implement an FFQ to estimate the consumption of traditional locally harvested foods for Dene/Metis in the Northwest territories, Canada’. *Rationale for tool validation*: To better understand the public health challenges posed by contaminants in traditional foods	FFQ Newly developed tool 319 questions on 61 food items Frequency with pictorial guide for serve size quantification 12‐month recall period Self‐administered by smart tablet Culturally relevant foods included English language	Frequency of intake and serve size No nutrition intake quantification	Face and content validity	Focus Groups	Confirmed useability and that food list is acceptable to target population
38	Schulz et al. [35]	*n* = 21 Pima Indian adults 12 Men and 9 Women Mean age 31.3/35.4 (M/F) Gila River Indian Reservation Arizona, USA	‘To assess the validity of methodology applied to evaluate dietary intake and physical activity in large‐scale surveys of the Pima Indians of Arizona’ *Rationale for tool validation*: Chronic disease prevention	Labelled as a FFQ in the article but describes the tool using a DHQ format also references the pre‐existing Burke DHQ Assesses usual intake Not reported if adapted or modified in any way. Interviewer‐administered English language	Energy (MJ) % total energy from Carbohydrate Fat Protein Alcohol	Relative validity	Two weeks of daily sequential 24‐h dietary recalls immediately post FFQ administration	Significant relationship between Energy intake measured by FFQ and total energy expenditure as measured by doubly labelled water. No significant correlations between FFQ and anthropometric measurements Authors state that the two methods produced similar results however on an individual basis results ‘appeared less reliable’ in those with high alcohol consumption.
39	Simmons et al. [36]	*Multi‐ethnic sample* *n* = 367 New Zealand adults 127 Europeans 103 Māori 167 Pacific Islander 33% Male Mean age of 36	To design and validate a rapid and simple tool to assess the impact of a diabetes awareness programme in South Auckland. OF particular importance is that the questionnaire was understandable, easy to use and culturally appropriate for a population that includes a high proportion of Polynesians or both Māori and Pacific Island descent *Rationale for tool validation*: Chronic disease prevention	FFQ Newly developed tool 12‐item Interviewer‐administered English language with interpreter offered but never required Not further specified	Energy (kcal)	Construct validity Reproducibility Relative validity	High Fat/High Refined Carbohydrate Score (HFHRC) Fat Index	Pearsons r for energy intake as determined by FFQ versus **HFHRC**: *r* = 0.38 (Māori, *p* < 0.01), *r* = 0.44 (Pacific Islander, *p* < 0.001) **Fat Index**: *r* = 0.26 (Māori, *p* < 0.05), *r* = 0.05 (Pacific Islander, not significant) Pearsons r for reproducibility of the FFQ was *r* = 0.45 (Māori, *P* < 0.01), *r* = 0.52 (Pacific Islander, *p* < 0.001) Pearsons *r* for energy intake as derived from FFQ compared to Dietetic assessments was *r* = 0.62 (Māori, *p* < 0.001), *r* = 0.65 (Pacific Islander, not significant)
40	Slattery et al. [37]	*n* = 6604 American Indian/Alaska Native adults from Alaska and Southwest USA 64% female Aged 18–65+ years	‘To develop a self‐administered computer‐assisted comprehensive DHQ to collect dietary intake data for a prospective study of American Indian and Alaska Natives. One of the goals in developing the DHQ was to be comprehensive yet sensitive to unique dietary patterns that exist among American Indian and Laska Native tribes’ *Rationale for tool validation*: To understand the scope of dietary associations with disease	EARTH – DHQ Newly developed 70+ items (54 main food group questions with questions on preparation method) Pictorial guide for serve size quantification using default values on serve size 12‐month recall period Self‐administered via computer programme English language Translated into local language Included culturally relevant foods	Core Food Groups Energy (kcal) Frequency of foods	Acceptability	Parametric data	Complete data for almost 100% participants Tool can be completed by most study participants with minimal time and assistance. The respondent burden was reasonable Authors report the methods developed for collection of dietary data appear to be appropriate for the targeted population Energy intake derived from FFQ fell within acceptable range for 79%–82% or participants
41	Smith et al. [38]	*n* = 525 Pima Indian adults 273 Men and 302 Women, aged 18–74 years From the Gila River Reservation Arizona, USA	‘To determine the usual intake on the Gila River Indian community in Arizona using two methods of dietary assessment – 24‐h recall and quantitative food frequency questionnaire’ *Rationale for tool validation*: Chronic disease prevention	Labelled as a FFQ in the article but describes the tool using a DHQ format also references the pre‐existing Burke DHQ Assesses usual intake Not reported if adapted or modified in any way. Interviewer‐administered English language. Same tool as article # 38 above however different validation method described	Energy (kcal) **Macronutrients** Carbohydrate [total] (g) Fat [total, saturated, monounsaturated, polyunsaturated] (g) Protein [total] (g) Cholesterol (mg) Fibre [total, soluble] (g) **Micronutrients** Ca (mg) Fe (mg) Na (mg) *Vitamins* A (IU) Beta‐carotene (μg) C (mg)	Relative validity Test–retest reliability Inter‐rater reliability	1× 24‐h dietary recall administration contemporaneous with FFQ administration Repeat administration by same interviewer Repeat administration by different interviewer	Paired *t*‐test and Spearman correlations for each food domain stratified by sex showed statistically significant differences between the FFQ and 24‐h recall with Spearman correlation coefficients ranging *r* = 0.2–0.41 for macronutrients, *r* = 0.36–0.47 for micronutrients and *r* = 0.17–0.36 for vitamins Intraclass correlations (ICC) = 0.72 for energy, 0.65 for protein, 0.63 for carbohydrate, 0.75 for fat Intraclass correlations (ICC) = 0.76 for energy, 0.68 for protein, 0.76 for carbohydrate, 0.69 for fat
42	Wilkens et al. [39]	*Multi‐ethnic sample* *n* = 352 77 African American 62 Japanese American 71 Latino American 69 Native Hawaiian 73 White Male (49.2%) and Female (50.9%) Aged 55–80 years from Hawaii and Los Angeles, USA	‘To compare nutrient intakes estimated from two different QFFQ each other and to intakes calculated from three 24‐h dietary recalls (24HDRs)’ *Rationale for tool validation*: Understanding how updated instruments used for a large scale study agree with each other	**Baseline QFFQ** Pre‐existing tool 180‐item Frequency with serve size quantification 12‐month recall period Self‐administered Included culturally relevant foods English **10‐year QFFQ** Newly developed tool 180‐item Frequency with serve size quantification 12‐month recall period Self‐administered Included culturally relevant foods English	Energy (kcal) **Macronutrients** Carbohydrate [total] (g) Fat [total, saturated] (g) Protein [total] (g) Fibre (g) **Micronutrients** Ca (mg) *Vitamins* A (μg) C (mg)	Relative validity	3× 24 h recalls (2 weekday and 1 weekend day) over 1 month via telephone by Dietitians using the Automated multiple pass method	Significant correlations (*p* < 0.05) between the Baseline QFFQ and 24 h recalls for Native Hawaiians: Energy *r* = 0.35 Macronutrients: 0.23–0.53 except protein and saturated fat, which were not significant Minerals: *r* = 0.32 Vitamins: *r* = 0.32–0.44 except beta‐carotene, which was not significant Increased significant correlations (*p* < 0.05) between the 10‐year QFFQ and 24 h recalls for Native Hawaiians: Energy *r* = 0.67 Macronutrients: 0.44–0.65 (all significant) Minerals: *r* = 0.43 Vitamins: *r* = 0.14–0.35 except beta‐carotene and Vitamin A which were not significant Native Hawaiian cohort had highest amount of nutrient correlation findings that did not reach statistical significance threshold than other groups among the multiethnic cohort (*n* = 9 vs. 0–7)

Abbreviations: Ca = calcium, DHA = docosahexaenoic acid, DHQ = diet history questionnaire, EEE = estimated energy expenditure, EEI = estimated energy intake, EPA = eicosapentaenoic acid, Fe = iron, FFQ = food frequency questionnaire, I = iodine, ICC = intraclass correlation, K = potassium, kJ = kilojoule, Mg = magnesium, MJ = megajoule, Na = sodium, NSW = New South Wales, *p* = phosphorous, QLD = Queensland, SA = South Australia, Se = selenium, WA = Western Australia, Zn = zinc.

### 2.4 CREATE Quality Appraisal Tool (QAT)

The CREATE QAT [14] assessed the adherence of included studies' methods against gold‐standard Indigenous research principles. The QAT was designed by Aboriginal and Torres Strait Islander researchers within Australia for the purpose of assessing the cultural appropriateness, relevance and quality of research conducted with Aboriginal and Torres Strait Islander communities through an Aboriginal and Torres Strait Islander lens [40]. The QAT assesses several domains including governance, Indigenous research paradigm, cultural and intellectual property, and capacity strengthening [40]. For the purposes of this review, the tool was adapted for Indigenous populations globally. The domains assessed for research pertaining to Aboriginal and Torres Strait Islander peoples on the QAT are consistent with gold‐standard research principles for international Indigenous population groups [41] and was therefore expected to perform cross‐culturally. Appraisal with the QAT was conducted simultaneously by the lead author (MK) and an Aboriginal researcher (VW). Classification of scores is not outlined within the QAT guide [40]; however, scores within this review have been classified in line with similar existing literature [7] for ease of comparison as follows; number of ‘yes’ or ‘partially’ scored items: 1–5 = low, 6–9 = moderate and 10–14 = high.

### 2.5 Data Synthesis

Results are presented in tabular and graphical format with narrative synthesis to address the research questions. Descriptive statistics and linear regression analysis were performed to analyse QAT score trends over time in *R*+ Studio 2023.6.1.524 [42]. All other graphs were generated within Excel, including heatmapping the mean correlation strength of relative validity between the tools and chosen reference method for select foods and nutrients.

## 3 Results

**FIGURE 1:**
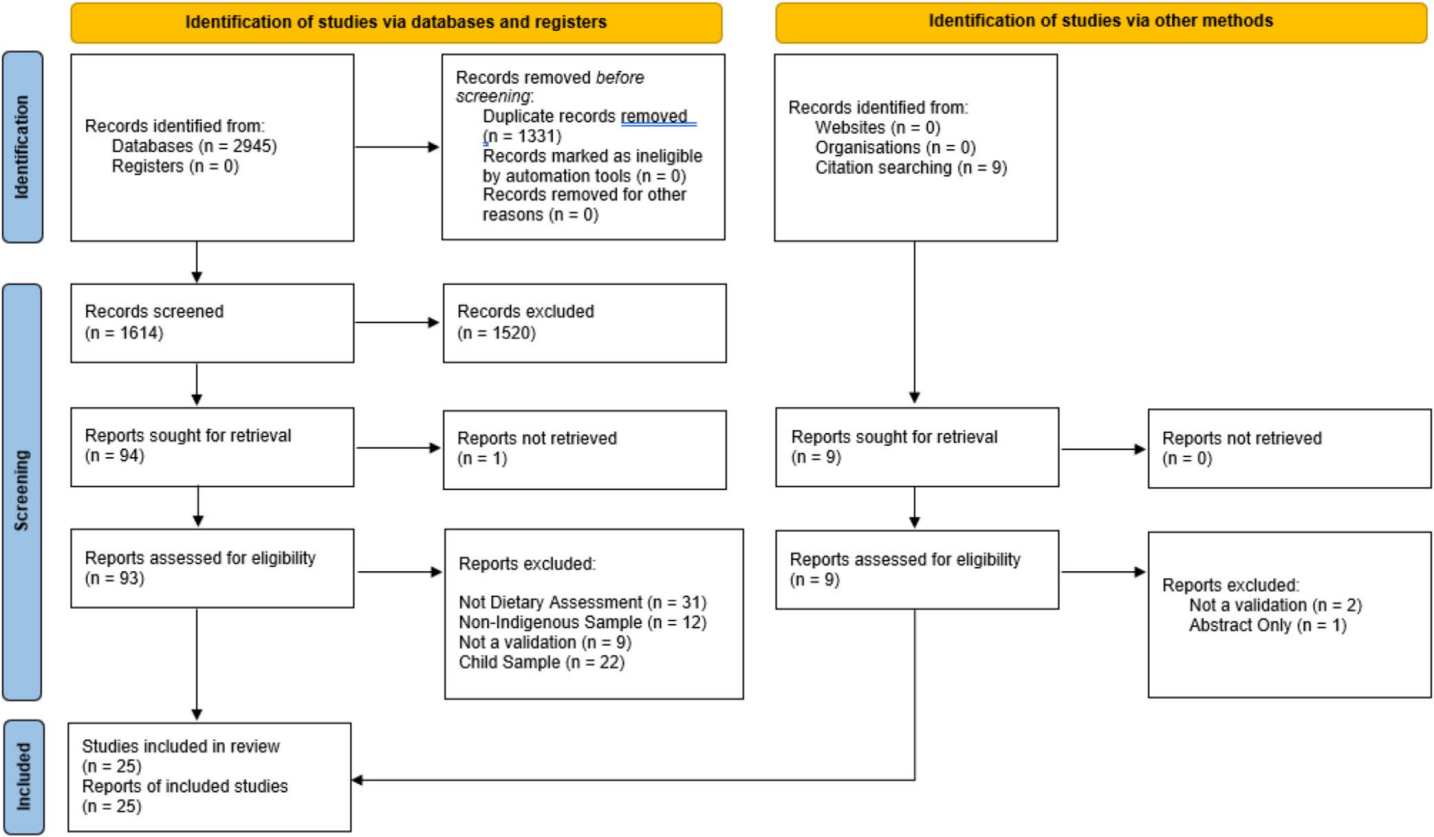
PRISMA diagram.

After exclusions there were 25 studies [15, 16, 17, 18, 19, 20, 21, 22, 23, 24, 25, 26, 27, 28, 29, 30, 31, 32, 33, 34, 35, 36, 37, 38, 39] included in this review (Figure 1), describing 31 separate tool validations. Four studies validated more than one tool simultaneously [19, 22, 30, 39], and seven reported more than one type of validity or reliability testing [17, 20, 27, 28, 32, 36, 38]. Several tools were the subject of multiple studies being validated in different Indigenous populations. Ultimately there were 28 unique tools validated among the included studies. Study characteristics are detailed in Table 2. Most studies were conducted in the United States with American Indian/Alaska Native (AI/AN) and Native Hawaiian populations (*n* = 13, 52%) followed by Aboriginal and Torres Strait Islander populations in Australia (*n* = 4, 16%), Indigenous peoples of Canada (*n* = 3, 12%), Inuit populations in Greenland (*n* = 3, 12%) and Māori and Pacific Islander populations in New Zealand (*n* = 2, 8%). Sample sizes ranged from 15 to 6604 participants; 75% of included studies had a sample size less than *n* = 206 Indigenous participants. Larger sample sizes were seen in one retrospective validation of national survey methods [19], and one large‐scale validation study from the USA [37]. Most studies included male and female participants in their sample; however, 20% (*n* = 5) were restricted solely to women or pregnant women. Aside from six studies that sampled those aged > 40 years [15, 16, 23, 28, 31, 39], studies included a wide range of ages of adult participants(18–88 years).

**TABLE 2:** Characteristics of studies and self‐report dietary assessment tools that have undergone validation for Indigenous populations globally.

Study characteristics		Tool characteristics	
**Populations/Location**	*n*	**Novelty**	*n*
American Indian/Alaska Native (USA)	11 [18, 21, 22, 24, 25, 29, 30, 31, 35, 37, 38]	Newly developed tool	16 [15, 16, 17, 20, 21, 23, 24, 25, 27, 30, 31, 32, 34, 36, 37, 39]
Indigenous (Canada)	3 [32, 33, 34]	Existing tool	15 [18, 19, 22, 26, 33, 35, 38, 39]
Aboriginal or Torres Strait Islander (Aus)	4 [15, 17, 26, 27]	**Type of Tool**	
Pacific Islander/Native Hawaiian (USA)	2 [23, 39]	Food frequency questionnaire	16 [15, 16, 18, 20, 22, 25, 26, 28, 30, 31, 32, 33, 34, 36, 39]
Māori or Pasifika (NZ)	2 [28, 36]	Diet history questionnaire	6 [21, 23, 29, 35, 37, 38]
Inuit (Greenland)	3 [16, 19, 20]	Survey	6 [19, 24, 27]
		Diet record	1 [17]
		Inventory	1 [30]
**Sample size**	*n*	**Number of items**	
Min	15	Min	2
Max	6604	Max	319
Mean	728	Mean	79
Median	91	Median	51
**Participants age**	*years*	**Period assessed**	*n*
Range	18–88	12 Months	13 [16, 20, 21, 22, 23, 25, 27, 29, 31, 34, 37, 39]
Mean	42.6	3 months	1 [28]
**Gender**	*n*	1 month	5 [15, 18, 30, 32, 33]
Both male and female	20 [15, 16, 19, 20, 21, 22, 23, 25, 26, 27, 28, 31, 32, 33, 34, 35, 36, 37, 38, 39, 43]	Usual intake	7 [19, 24, 26, 35, 38]
Exclusively male	0	Past season/most recent intake	4 [19, 22, 27, 30]
Exclusively female	2 [29, 30]	Unreported	2 [17, 36]
Female – Pregnant	3 [17, 18, 24]	**Method of administration**	*n*
**Validity assessed**	*n*	Interviewer	14 [15, 16, 20, 23, 25, 26, 30, 31, 32, 33, 35, 36, 38]
Concurrent/Relative validity	23 [16, 18, 19, 20, 22, 23, 24, 27, 28, 29, 30, 31, 32, 33, 35, 36, 38, 39]	Self	16 [17, 18, 19, 21, 22, 24, 27, 28, 29, 30, 34, 37, 39]
Construct validity	1 [36]	Both Interviewer‐ or Self—	1 [22]
Face validity/Acceptability	6 [15, 17, 21, 25, 26, 34]	Modality	
**Reliability assessed**	*n*	Paper‐based	24 [16, 18, 19, 20, 22, 23, 24, 25, 26, 28, 30, 31, 32, 33, 35, 36, 38, 39]
Test–retest reliability	6 [20, 27, 28, 29, 36, 38]	Computer software	4 [15, 21, 29, 37]
Inter‐rater reliability	2 [17, 38]	Device app	3 [17, 27, 34]
		Browser	0
		**Language**	*n*
		English	25 [15, 17, 18, 21, 22, 23, 24, 25, 26, 27, 28, 29, 30, 31, 32, 33, 34, 35, 36, 37, 38, 39]
		Danish	6 [16, 19, 20]
		Local Interpreter	8 [16, 20, 25, 26, 31, 32, 33, 36]
		Translated local language	8 [19, 21, 27, 29, 37]

Of the 31 tool validations (Table 2), 15 were existing tools being newly applied within an Indigenous population, with or without adaptation, and 16 were newly developed tools undergoing initial validation. The most validated tool type was Food Frequency Questionnaires (FFQs, *n* = 16), followed by Diet History Questionnaires (DHQs, *n* = 6) and surveys (*n* = 6). One study each used a diet record and inventory method. The most common method of validation reported was relative validity (*n* = 23). One study [17] assessed the relative validity of the tool and purposively sampled Aboriginal and Torres Strait Islander participants, but only reported the data separately for acceptability; therefore, the relative validity component was ineligible for inclusion in this review. This study had *n* = 25 participants in the total sample, including *n* = 8 Aboriginal participants, which may explain why analysis was not reported separately, although this could not be confirmed. Eight reported on reliability testing, the most common form being test–retest reliability (*n* = 6), followed by inter‐rater reliability (*n* = 2).

There were 24 paper‐based tools and 7 digital tools (*n* = 4 computer and *n* = 3 smart device‐based apps) validated among the included studies. Of these, interviewer‐administered paper tools (*n* = 13, 42%) were the most common tools that underwent validation with Indigenous populations, followed by self‐administered paper (*n* = 10, 32%), computer software (*n* = 3, 10%), smart device app (*n* = 3, 10%)‐based tools and interviewer‐administered computer software‐based tools (*n* = 1, 3%). One paper‐based tool had the option to either self‐ or interviewer‐administer [22]. There were no web‐browser‐based dietary assessment tools in the included studies. Tools most commonly assessed a recall period of 12 months (*n* = 13, 42%), followed by usual intake (*n* = 7), 1 month (*n* = 5) and 3 months (*n* = 1).

**FIGURE 2:**
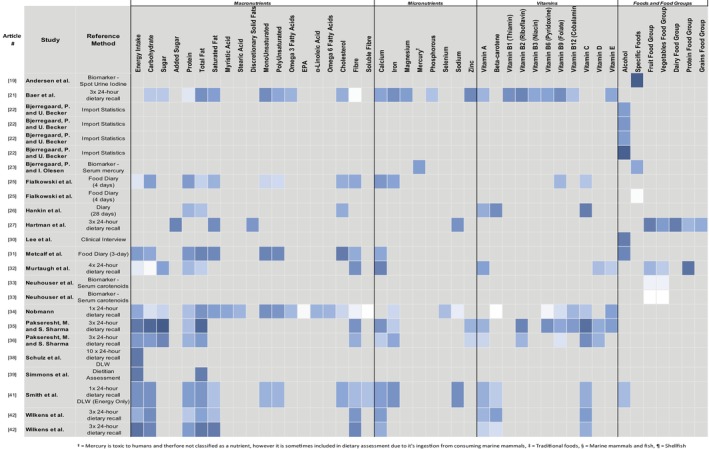
Heatmap of reported correlation co‐efficients between selected food and nutrient domains and the repsective reference method within relative validity studies of self‐report dietary assessment tools among Indigenous population groups globally.

Tools were primarily delivered in English (*n* = 25, 81%), with 26% (*n* = 8) also utilising an interpreter for local languages during administration, and 26% (*n* = 8) pre‐translating the tools (written or audio translation). It was 1.6 times as common for interviewer‐administered tools to report providing an interpreter compared to the translation of self‐administered tools (Figure 2). Four of the six self‐administered digital tools provided translation utilising audio recording in the app or computer software [21, 27, 29, 37], and none reported providing text translation. Text translation of paper tools was provided in one study [19].

**FIGURE 3:**
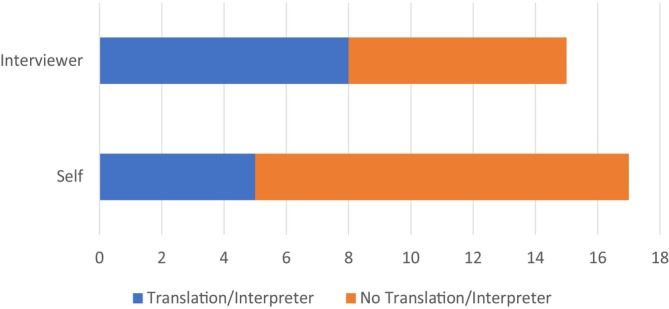
Provision of language translation by tool administration method.

Relative validity was undertaken on 23 tools that assessed between 1 and 26 food and nutrient domains with a mean of 10 domains assessed. Correlation strength findings (Figure 3) between the tools and their respective reference method for each study that reported on relative validity findings, with darker blue indicating stronger correlation results. Studies validated for total fat (*n* = 12, 50%), energy and protein (*n* = 11, 46% each) and carbohydrate (*n* = 10, 42%) more often than any other domains.

The most common reference method used to assess the relative validity of tools was 24‐h dietary recalls (*n* = 10, 42%). Correlation coefficients ranged from *r* = 0.00–0.82 (Figure 3). Within the studies that reported p‐values, all correlations *r* > 0.3 were statistically significant (*p* < 0.05) except for the study by Hankin et al.(1991), with correlations for vitamin A, vitamin C and fibre not statistically significant [23]. Wide variations in strength of association can be seen within each food and nutrient domain; however, more defined trends are apparent within individual studies. Macronutrients generally achieved stronger correlation than micronutrients and vitamins.

### 3.1 QAT Results

**FIGURE 4:**
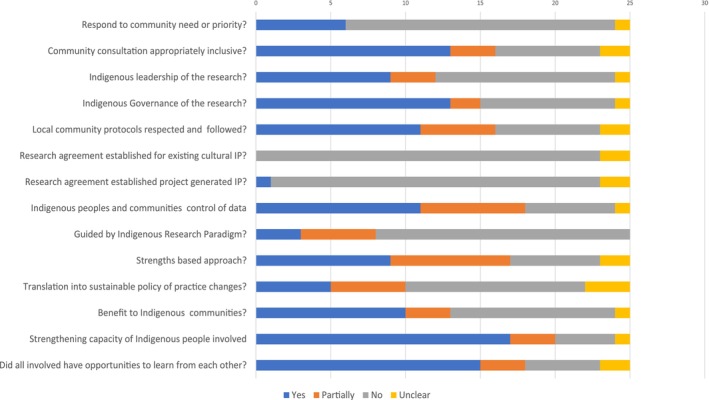
Distribution of scores across QAT items for studies validating self‐report dietary assessment tools for Indigenous populations globally.

A third of the studies each scored highly and moderately on the QAT (*n* = 8, 32% each respectively), and 36% rated as low (*n* = 9). There was a slight trend for studies to score higher on the QAT over time in linear regression analysis; however, this was not significant (*p* = 0.144). The most frequently reported items among the studies (Figure 4) were capacity strengthening for Indigenous individuals involved in the study (*n* = 20), Indigenous peoples having control over the collection and management of data (*n* = 18), and all those involved in the study having opportunities to learn from each other (*n* = 18). The least frequently reported item was studies reporting to have negotiated agreements to protect Indigenous peoples intellectual or cultural property, with this reported in one study [27], responding to community needs and being guided by Indigenous research principles (*n* = 6 and 8, respectively). Studies that scored low on the QAT performed no differently in terms of correlation with the respective reference method than those that scored highly on the QAT in regression analysis (*p* = 0.207).

## 4 Discussion

This scoping review aimed to identify and examine the scope of self‐report dietary assessment tools and their validation methods for Indigenous adult populations globally, for the purpose of informing the development of a self‐report dietary assessment tool for an Indigenous population in Australia. This review identified 28 dietary assessment tools that had undergone some form of validity or reliability testing within Indigenous adult population groups, with several tools being the subject of more than one study, resulting in 31 validations reported worldwide.

The authors recognise that Indigenous population groups globally are unique and diverse and therefore findings are not necessarily generalisable cross‐culturally. There are, however, shared experiences of western colonisation among Indigenous population groups such as traditional subsistence lifestyles, dispossession of Indigenous peoples of their ancestral lands, medicines, food systems and waterways and propensity to live more remotely [44, 45]. Indigenous population groups within Australia, Canada and New Zealand also experience comparable political structures for those countries remaining in the Commonwealth [3]. Therefore, incorporating and synthesising international literature enables insights to be shared for the benefit of any new endeavours into the field at large. Findings from this review should be considered with this in mind and applied with due consideration of the local Indigenous context.

Dietary assessment methods are often euro‐centric in their design. Davies et al. (2023) recommend the exploration of Indigenous research methods such as yarning to improve the efficacy of dietary assessment used in research for Aboriginal and Torres Strait Islander peoples [7]. However, where resources or accessibility may be limited, or quantification of individual‐level dietary intake is required, the need for portable, low‐cost self‐report dietary assessment methods will persist. This review found that within this category, FFQs were the predominant tool format validated within Indigenous populations globally and achieved varying degrees of validity. Whilst small sample sizes (*n* < 30) may have affected the statistical power of findings in several studies, this is outside the scope of this review to critique.

### 4.1 Tool Characteristics

FFQs have been found to have low applicability within Aboriginal and Torres Strait Islander populations [7], but were frequently utilised due to portability, cost and reduced participant and administrator burden [15, 16, 18, 27, 28, 31, 32, 33, 34]. There were arguments made among the included studies that, despite accuracy issues for FFQs, their utility in Indigenous population groups outweighs the risk of bias if measures are taken to support accurate reporting. This involves accounting for a wide range of literacy diversity, inclusion of locally and culturally relevant foods, sufficient partnership and consultation to ensure cultural appropriateness, and understanding cultural interpretations of food and nutrition. A sentiment echoed by Willet and Hu (2006) who acknowledge that a single FFQ will not be applicable in all populations [46]. They argue that building contextually appropriate FFQs is a worthy endeavour that will maximise efficacy rather than a burden that justifies abandoning the FFQ entirely as a dietary measure [46].

Although half the tools were developed specifically for Indigenous populations, not all provided an interpreter or translation of the tool into the local language. When provided, it was more commonly in‐person interpreters than written translations. As there were almost equal numbers of interviewer and self‐administered tools, this could be due to many Indigenous languages being spoken rather than written [9], which may necessitate the use of interpreters over text translation. The provision of interpreters can also be costly and logistically challenging to provide in geographically isolated communities [47, 48], where most of the included studies took place. Device‐based e‐tools commonly provided audio recorded translations, effectively minimising the barrier to translation of self‐administered tools whilst avoiding the financial and administrative burden associated with interpreters.

Despite the rapid advancement of health technologies over the last three decades, less than 20% of the tools in this review were computer or smart device‐based (e‐tools). This may be due to feasibility concerns around historically lower rates of personal computer ownership experienced by Indigenous peoples, particularly those living remotely [43]. This barrier to accessibility is then further compounded by the need for standalone programmes that may be difficult to acquire or require training. The advent of the smartphone has opened up novel options for health information and assessment delivery, such as e‐tools hosted within a web browser, accessible by personal smart device, that do not require dedicated software to be downloaded or purchased. There was a distinct lack of browser‐based e‐tools, which are emerging in the field and have been shown to perform comparably to other formats of dietary assessment for Australian Indigenous children [49]. Device‐based tools in general also have the potential for functionality and language translation that circumvents barriers associated with paper‐based tools whilst maintaining portability, often for lower cost and participant and administrator burden [27, 43]. The studies in this review that utilised device apps performed face validity and acceptability rather than relative validity, except for the Grog App [27], which solely assessed alcohol intake and no other food or nutrient domains. Consequently, trends for how differing types of e‐tools correlate to established reference methods were unable to be analysed in this review.

Despite a history of lower computer ownership, there has been a significant uptake of smartphone use within Indigenous populations over time [43]. A recent Australian survey exploring Aboriginal women's access to and interest in mHealth (medical and public health activities supported by personal mobile devices) found high mobile platform engagement and acceptance and interest in mHealth solutions [50]. These solutions are uniquely placed to provide health assessment or advice where access to primary health or health promotion can be limited, such as in rural and remote Indigenous communities. However, caution needs to be exercised on the availability and reliability of technology within Indigenous populations, particularly in rural or remote areas where signal coverage may be compromised, to ensure digital inclusion [43, 50].

### 4.2 Validation Methods

Despite the rationale provided for pursuing validation of these tools with Indigenous populations often being chronic disease prevention (Table 1) several food or nutrient domains associated with chronic disease risk, such as added sugar, sodium and core food groups, were not commonly assessed (*n* = 1 and *n* = 3 studies, respectively). The most common domains were total energy and macronutrient (fat, protein and carbohydrate) intakes. The dominance of these domains is likely due to their ability to be validated against multiple different reference methods and established links to health outcomes compared to some micronutrients, of which intake may be more variable. However, chronic disease prevention and health promotion programmes intended for Indigenous populations typically include education and strategies to increase fruit and vegetable intake and reduce the consumption of food and drinks high in sugar and salt [51, 52, 53]. Designing and validating tools to discern intake of these domains may improve the sensitivity of tools to dietary behaviour change and enable more meaningful measurement of health promotion outcomes for Indigenous peoples.

Wide variations in reference methods used for relative validity were found among the studies. It was common for studies to use alternate reference methods than the gold standards, which prioritise objective over subjective methods. Total energy intake was one of the most common attributes validated for, yet 2 of the 11 studies [35, 38] utilised the established gold standard objective reference method of doubly labelled water to assess its correlate of total energy expenditure [10]. Instead, studies validated against other subjective methods such as food diaries and 24‐h recalls. Weighed food records are the preferred reference method when validating against other dietary recall measures [11]; however, this was not used as a validation measure among the included studies. Such variation in methods suggests that studies validating dietary assessment methods among Indigenous populations are accounting for and prioritising the contextual and/or cultural appropriateness of research methods in their research design. As explained by Cade et al. 2002, reference method suitability is dependent on participant demographics and characteristics [11]. When accommodating participants with English as a second language or other types of literacy diversity—as may be relevant with Indigenous populations—24‐h dietary recalls may be more appropriate due to lower participant burden [11]. This is further supported by Davies et al. (2023) who found WFRs to be inappropriate for Aboriginal and Torres Strait Islander populations in Australia [7]. Therefore, the high utilisation of these methods over the established objective gold standards for dietary assessment methods in this context is expected.

### 4.3 Reporting in Multiethnic Samples

Within the studies with multiethnic samples, the correlation coefficients between measures from the tool and reference method for Indigenous groups were consistently lower and achieved less statistical significance than the non‐Indigenous groups [18, 23, 28, 39]. Indigenous peoples were often the minority within the sample groups. This highlights the importance of reporting correlation data by ethnicity in multiethnic data sets inclusive of Indigenous populations. If analysed together, it is possible that Indigenous peoples' data are masked by the predominant data of non‐Indigenous peoples within the sample and may not reveal a tool's inadequate performance for Indigenous peoples. The risk is that the tools which do not perform equitably for Indigenous peoples will be used as a ‘validated measurements’ to evaluate health promotion or research outcomes, potentially misrepresenting dietary intakes.

### 4.4 QAT Findings

The rationale for utilising the QAT in this review was to scope the extent to which Indigenous research principles are being applied in dietary assessment tool validation for Indigenous population groups globally. In the absence of any known cross‐cultural alternatives, the QAT was adapted for this study. The QAT scores suggest that Indigenous research principles are being applied in studies validating dietary assessment methods; however, not uniformly across all items. Although capacity strengthening and learning opportunities were commonly positively scored, legal protections of Indigenous intellectual and cultural property, and responding to community need or priority were among the lowest scored items. This is consistent with the findings of Davies et al. (2023) who also reported no studies in their Australian review establishing research agreements regarding intellectual and cultural property [7]. However, it differed in that a majority of studies scored positively for research being guided by a community‐identified need or priority. This difference may be due to the review by Davies et al. scoping dietary assessment methods utilised in Indigenous research more broadly, as opposed to our review that was restricted to validation studies of tools, predominately used by researchers. A slight upward trend of QAT scores over time may reflect the evolution and increasing adoption of modern Indigenous research principles, and the QAT tool reflecting these modern expectations of Indigenous research [40, 53]. However, this trend was not significant nor consistent. Similarly, there were no statistically significant relationships between QAT ranking and the reported strength of relative validity. Due to the small sample size in this review, these results should be interpreted with caution.

### 4.5 Strengths and Limitations

This is the first review, to the authors' knowledge, exploring the range of self‐report dietary assessment tools that have been specifically validated for use with Indigenous populations globally. Nonetheless, this review does have limitations. Despite some shared experiences of western colonisation, Indigenous population groups are diverse and unique. Therefore, findings are not necessarily generalisable cross‐culturally, and the application of the findings of this review should be considered within local contexts. The QAT is a subjective measure and is not immune to bias. To address this, an Aboriginal Researcher (VW) provided guidance on this review and duplicated QAT scoring with the lead author to ensure Indigenous perspectives were included and to minimise potential bias. Small sample sizes may have contributed to the lack of significant trends found within the linear regression results of the QAT scores and association with validation findings in a smaller subset of relative validity articles.

## 5 Conclusions and Implications

Interviewer‐administered FFQs are the most prevalent self‐report dietary assessment method validated for use with Indigenous populations globally. There is an apparent misalignment between food and nutrient domains used to determine tool validity and the domains that most align with outcome measures commonly targeted by chronic disease prevention programmes with Indigenous populations. This is despite chronic disease prevention being the predominant catalyst for health promotion programmes within Indigenous populations globally. In the pursuit of Indigenous health equity, ensuring tools are adequately capturing these dietary domains may provide for a more meaningful evaluation of health promotion programmes. Browser‐based e‐tools which are portable and cost‐effective may hold promise for dietary assessment among Indigenous populations. The acceptability and validity of such tools for Indigenous population groups should be explored through future research.

## Ethics Statement

Ethical approval was not sought for this review as it wholly utilised the existing published literature.

## Conflicts of Interest

The authors declare no conflicts of interest.

## Data Availability

Data sharing is not applicable to this article as no new data were created or analyzed in this study.

## Appendix A

### Medline Search Strategy

**Table:**

#	Search terms	Results
1	exp indigenous peoples/	35795
2	((“first nation*” or “indigenous people*” or “indigenous population*” or “native born” or “native people*” or “native‐born” or natives or “american native continental ancestry group” or “american amerind*” or “american indian*” or “north american amerind” or “north american amerinds” or “north american indian” or “north american indians” or “canadian native*” or “indigenous canadian*” or “metis canadian*” or “native canadian*” or aleut* or eskimo* or inuit* inupiat* or kalaallit* or Inuvialuit or “american indian*” or “alaska native*” or “american native*” or “native american*” or “alaska indigenous people*” or “alaska's indigenous people*” or “alaskan native*” or “alaskas indigenous people*” or “indigenous alaska*” or “indigenous people of alaska” or “native alaskan*” or “aboriginal australian*” or “indigenous australian” or aborigin* or “australian race” or “Torres Strait Islander*” or “Canadian Indian*” or “Canadian Aboriginal* australoid” or maori or “native hawaiian*” or “Hawaii native*” or “hawaiian native*” or “pacific islander*” or “oceanic ancestry group*” or “pacific island american*” or sami or yupik or Mapuche or indigenous or Aymara or Tsimane or “south sea islander*”).mp. [mp=title, book title, abstract, original title, name of substance word, subject heading word, floating sub‐heading word, keyword heading word, organism supplementary concept word, protocol supplementary concept word, rare disease supplementary concept word, unique identifier, synonyms])	84355
3	1 or 2	94392
4	exp diet/	324367
5	((diet* or nutrition* or food) and (assess* or tool* or measur* or survey or questionnaire*)).mp. [mp=title, abstract, heading word, drug trade name, original title, device manufacturer, drug manufacturer, device trade name, keyword heading word]	557531
6	exp validation study/	109105
7	(valid* or reliab*).mp. [mp=title, abstract, heading word, drug trade name, original title, device manufacturer, drug manufacturer, device trade name, keyword heading word]	1443135
10	6 or 7	1443153
11	4 or 5	768600
12	3 and 10 and 11	336
